# *In Silico* Analysis of Phosphomannomutase-2 Dimer Interface Stability and Heterodimerization with Phosphomannomutase-1

**DOI:** 10.3390/molecules30122599

**Published:** 2025-06-15

**Authors:** Bruno Hay Mele, Jessica Bovenzi, Giuseppina Andreotti, Maria Vittoria Cubellis, Maria Monticelli

**Affiliations:** 1 Biology Department, University of Naples Federico II, Complesso Universitario Monte Sant’Angelo, Via Cinthia, 80126 Naples, Italy; 2 Institute of Biomolecular Chemistry ICB, National Research Council of Italy, Via Campi Flegrei 34, 80078 Pozzuoli, Italy

**Keywords:** structural bioinformatics, PMM2-CDG, human PMMs, PPI

## Abstract

Phosphomannomutase 2 (PMM2) catalyzes the interconversion of mannose-6-phosphate and mannose-1-phosphate, a key step in the biosynthesis of GDP-mannose for N-glycosylation. Its deficiency is the most common cause of congenital disorders of glycosylation (CDGs), accounting for the subtype known as PMM2-CDG. PMM2-CDG is a rare autosomal recessive disease characterized by multisystemic dysfunction, including cerebellar atrophy, peripheral neuropathy, developmental delay, and coagulation abnormalities. The disease is associated with a spectrum of pathogenic missense mutations, particularly at residues involved in dimerization and catalytic function (i.e., p.Phe119Leu and p.Arg141His). The dimerization of PMM2 is considered essential for enzymatic activity, although it remains unclear whether this supports structural stability alone, or whether both subunits are catalytically active—a distinction that may affect how mutations in each monomer contribute to overall enzyme function and disease phenotype. PMM2 has a paralog, phosphomannomutase 1 (PMM1), which shares substantial structural similarity—including obligate dimerization—and displays mutase activity *in vitro*, but does not compensate for PMM2 deficiency *in vivo*. To investigate potential heterodimerization between PMM1 and PMM2 and the effect of interface mutations over PMM2 dimer stability, we first assessed the likelihood of their co-expression using data from GTEx and the Human Protein Atlas. Building on this expression evidence, we modeled all possible dimeric combinations between the two paralogs using AlphaFold3. Models of the PMM2 and PMM1 homodimers were used as internal controls and aligned closely with their respective reference biological assemblies (RMSD < 1 Å). In contrast, the PMM2/PMM1 heterodimer model, the primary result of interest, showed high overall confidence (pLDDT > 90), a low inter-chain predicted alignment error (PAE∼1 Å), and robust interface confidence scores (iPTM = 0.80). Then, we applied PISA, PRODIGY, and mmCSM-PPI to assess interface energetics and evaluate the impact of missense variants specifically at the dimerization interface. Structural modeling suggested that PMM2/PMM1 heterodimers were energetically viable, although slightly less stable than PMM2 homodimers. Interface mutations were predicted to reduce dimer stability, potentially contributing to the destabilizing effects of disease-associated variants. These findings offer a structural framework for understanding PMM2 dimerization, highlighting the role of interface stability, paralogs co-expression, and sensitivity to disease-associated mutations.

## 1. Introduction

**Figure 1:**
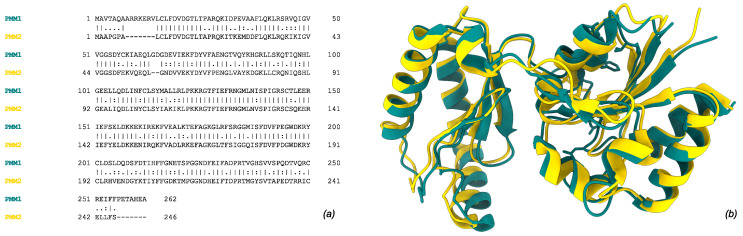
Human phosphomannomutase 1 (PMM1, tan, UniProt ID: Q92871) and 2 (PMM2, gold, UniProt ID: O15305). (**a**) Global pairwise alignment of the two paralogs using Needleman–Wunsch algorithm. (**b**) Structural superimposition of subunit crystal structures (2FUC for PMM1 and 7O4G for PMM2). Single, fully conserved residue “|”; groups of strongly similar properties—(>0.5 Gonnet PAM250) “:”; groups of weakly similar properties—(=<0.5 Gonnet PAM250) “.”.

Phosphomannomutases (PMMs, EC:5.4.2.8, InterPro: IPR005002) catalyze the isomerization of mannose-6-phosphate (Man-6-P) to mannose-1-phosphate (Man-1-P). In humans, this activity is potentially carried out by two paralogous enzymes, PMM1 and PMM2 [1], which share more than 60% sequence identity [2,3] and exhibit a conserved overall fold (RMSD ∼1 Å on crystallographic structures 2FUC for PMM1 and 7O4G for PMM2) (Figure 1). The formation of Man-1-P is a pivotal step in the biosynthesis of GDP-mannose and dolichol-phosphate-mannose, two sugar donors required for the formation of lipid-linked oligosaccharides [1,4].

The loss of PMM2 function leads to PMM2-CDG (OMIM: 212065; ORPHA: 793180), a congenital disorder of glycosylation (CDG) characterized by defective glycosylation [1,5,6]. PMM2-CDG is the most prevalent CDG subtype, with a reported prevalence as high as 1:20,000 [5]. The phenotypic spectrum of PMM2-CDG is highly variable, with predominant neurological manifestations, and disease severity ranges from severe neonatal-onset forms, which can be lethal (infant mortality rate of ∼20% within the first year of life), to milder phenotypes observed in adulthood [4,6,7]. Such variability is influenced by the diversity of pathogenic variants, which differentially affect protein folding, stability, and enzymatic activity, ultimately modulating disease progression and clinical outcomes [8]. Furthermore, the complete loss of PMM2 activity is incompatible with life, and pathogenic mutations are typically hypomorphic, thus preserving some residual function [9]. Most patients are compound heterozygotes, carrying one or more alleles that retain partial enzymatic activity [1,10].

Structurally, PMM2 functions as an obligate homodimer [1,11]. This suggests that *in vivo*, mixed dimers composed of different PMM2 variants may form, potentially influencing residual enzymatic activity depending on the specific mutational combination. These residual activities are modulated by various factors, including protein stability, folding efficiency, and integrity of the dimer interface. Indeed, the disruption of the dimeric assembly is increasingly recognized as a contributing factor to loss-of-function phenotypes. The most common genotype in Western Europe is p.Arg141His/p.Phe119Leu. While p.Arg141His is a mutation targeting the catalytic residue that impairs enzymatic activity, p.Phe119Leu disrupts homodimer formation, leading to protein destabilization and degradation [8,12].

PMM1, the closest paralog of PMM2, also forms an obligate homodimer and displays in vitro phosphomannomutase activity. Despite its structural similarity and enzymatic competence, PMM1 is not associated with any disease, and it is unable to compensate for PMM2 deficiency *in vivo* [13]. Interestingly, PMM1 has been identified as the primary bisphosphatase involved in the brain response to hypoxia, pointing out its peculiar role *in vivo* compared to PMM2 [14,15]. Given the primary involvement of the brain and central nervous system in PMM2-CDG, the role of PMM1 in this disorder, although still poorly understood, could be central and warrants further investigation.

The interconnection between PMM1 and PMM2 remains largely unexplored. In this work, we combined tissue-specific expression of PMM paralogs (based on GTEx and Human Protein Atlas), variant data mining, AlphaFold-based structural modeling of dimeric complexes, and mmCSM-PPI evaluation of dimers’ stability to explore the feasibility of PMM1/PMM2 dimerization and deepen the predicted effects of PMM2 variants on homodimers and heterodimers’ stability. Our findings provide new insights into the molecular features underlying PMM2 dimerization and heterodimer formation, with potential implications for understanding the biochemical basis of PMM2-CDG and its clinical variability.

## 2. Results

### 2.1. Tissue-Specific Expression of PMM Paralogs

**Figure 2:**
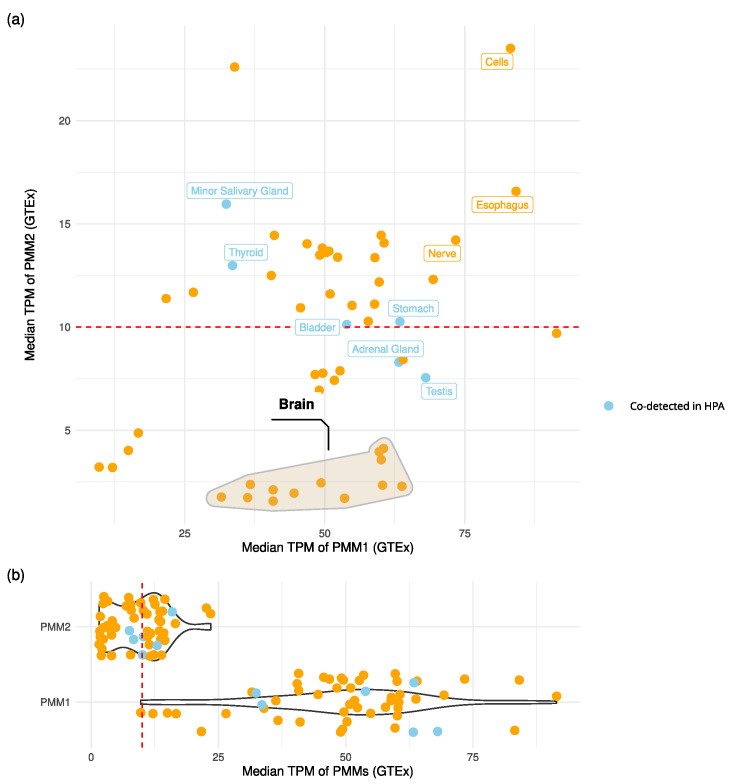
PMMs’ expression levels across tissues. (**a**) Scatter plot of *PMM1* vs. *PMM2* median transcript abundance (TPM) across GTEx tissues. Points associated with protein co-detection in the Human Protein Atlas (HPA) immunohistochemistry data are colored in light blue. The red dashed line marks the TPM threshold used to define robust transcript-level expression. The cluster of brain tissues is marked with a convex hull. Tissues with TPM > 10 for both genes and/or confirmed protein co-detection are labeled. (**b**) Violin plots of PMMs’ expression levels across tissues.

PMM1 and PMM2 are known paralogs, each capable of forming homodimers. Dual proteome-scale interaction networks suggest they interact via co-immunoprecipitation [16], hinting at the potential to form heterodimers. To evaluate the likelihood of such interactions occurring *in vivo*, we explored their co-expression and co-localization using GTEx (transcriptomics) and the Human Protein Atlas (HPA, proteomics) (Figure 2).

The analysis of GTEx transcriptomics data showed that co-expression at potentially functional levels (TPM > 10 for both genes) occurred in multiple tissues (Figure 2). Notably, esophagus mucosa, cultured fibroblasts, and tibial nerve tissues showed median expression values in the top quartile for both *PMM1* and *PMM2*. In the stomach, bladder and thyroid, HPA protein data confirmed GTEx expression patterns, indicating the presence of both proteins in the same tissues. Brain regions showed a distinct expression pattern: *PMM1* was consistently highly expressed (TPM > 25), while *PMM2* expression was below significance (TPM < 10). Despite this, statistically significant sample-level co-expression (Spearman $\rho >0.5;p_{\mathrm{adj}}\leq 0.05$) was observed in seven out of the thirteen brain subregions.

We attempted to validate the tissue-level co-expression of PMM1 and PMM2 using SpatialDB, the Human Reference Atlas Functional Tissue Unit Explorer, and the Allen Brain Atlas. However, none of these platforms included the specific tissues where we observed co-expression in bulk transcriptomic data, and in the available tissues, PMM1 and PMM2 did not appear to co-localize. Consequently, we retained GTEx and Human Protein Atlas as our primary sources for general co-expression patterns, while recognizing that future spatial or single-cell studies with deeper coverage will be necessary to resolve cellular co-localization.

### 2.2. Structure Evaluation

**Figure 3:**
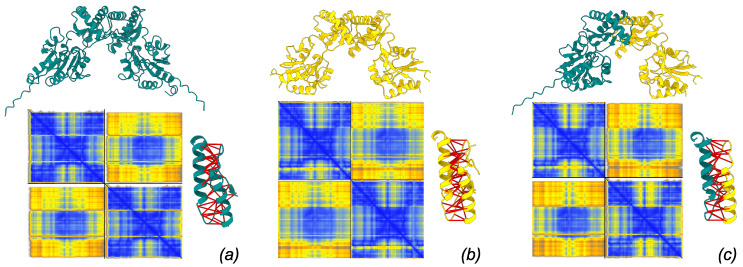
Models of dimer structure produced by AlphaFold3 server. Each model is associated with its PAE matrix (blue: low PAE, yellow: high PAE) and with the detail of interacting residues spanning ≤4 Å with predicted aligned error ≤5 Å (interactions marked by thick red pseudobonds). (**a**) PMM1 homodimer, (**b**) PMM2 homodimer, (**c**) PMM1/PMM2 heterodimer.

**Figure 4:**
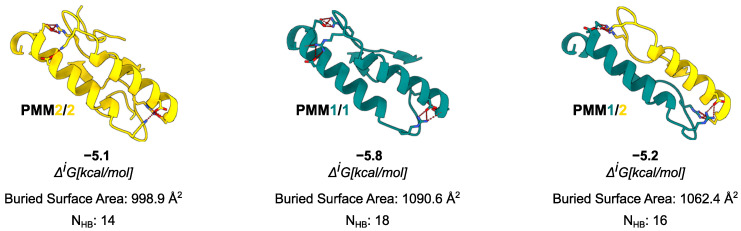
Comparison of interface composition and stability across the possible PMM dimers. For each dimer, the interface is shown as a cartoon, and residues involved in the formation of the most prominent salt bridges are displayed as sticks. Data from PISA analysis are reported in the figure.

Structural models of the PMM1 homodimer, PMM2 homodimer, and PMM2/PMM1 heterodimer were generated using AlphaFold3 in multimer mode (Figure 3). All three dimers showed highly similar overall fold and dimer architecture, consistent with the known crystal structures of PMM2 (PDB: 7O4G) and PMM1 (PDB: 2FUC) (Figure 4). The modeled dimers preserved the canonical “head-to-head” configuration typical of phosphomannomutases, and no major steric clashes or abnormal backbone conformations were observed upon inspection in ChimeraX.

Structural alignment between AlphaFold3 homodimer models and their respective crystal structures demonstrated strong agreement. For PMM1, the predicted dimer superimposed on the crystal structure 2FUC with an RMSD of 0.188 Å, while PMM2 aligned with 7O4G with an RMSD of 1.391 Å, in both cases, based on the alignment over the interaction CAP domain.

These low RMSD values, particularly the sub-angstrom deviation for PMM1, indicate high structural fidelity in the predicted interface. These results validate the capability of AlphaFold3 to accurately model the homodimeric interfaces of interest.

AlphaFold3 confidence metrics confirmed the robustness of the predicted interfaces. The PMM2 homodimer exhibited high confidence at the interface, with both chain and chain-pair iPTM scores of 0.81. The PMM1 homodimer showed a slightly lower chain iPTM score (0.78) and a marginally higher inter-chain PAE (∼0.89 Å), suggesting a somewhat less confident interface, though overall fold confidence remained high (chain pTM = 0.82). The PMM2/PMM1 heterodimer exhibited intermediate confidence, with chain iPTM values of 0.80, chain-pair iPTM values of 0.77–0.80, and an inter-chain PAE around 0.9 Å. These data suggest that all three dimers are structurally plausible, with minor differences in predicted interface stability.

### 2.3. Interface Analysis and Energetic Comparison

To benchmark the AlphaFold3-predicted complexes, we compared their interface features with available crystal structures of PMM1 (PDB: 2FUC) and PMM2 (PDB: 7O4G). The comparison between the AlphaFold3 model and the 7O4G (PMM2) crystal structure revealed the same number of interface residues (26), with a modest (∼100 Å^2^) difference in the interface area. Both models displayed a solvation free energy gain compatible with stable dimer formation $(\Delta^{i}G<-4$ kcal/mol). Similarly, the comparison between the AlphaFold3 model and the 2FUC (PMM1) crystal structure revealed the same number of residues (26), with an abysmal (∼30 Å^2^) difference in the interface area. Despite the low $\Delta^{i}$ (∼−1 kcal/mol, stable across other available crystals (2FUC, 6CFV)) reported by PISA for crystallographic PMM1 dimers, the literature consensus supports its obligate dimeric state based on symmetry, conservation, and biochemical evidence [17]. In contrast, AlphaFold3 models reconstructed a complete interface with $\Delta^{i}G=-5.8$ kcal/mol values consistent with stable dimer formation. The ∼−1 kcal/mol difference in $\Delta^{i}G$ between the crystallographic (7O4G) and AlphaFold-predicted dimers for PMM2 fell within the expected variability of PISA estimates and was not, by itself, indicative of a meaningful difference in interface stability.

Moving to the *in silico* analysis, the predicted PMM1 homodimer exhibited the largest interface area (1090.6 Å^2^), followed by the PMM2/PMM1 heterodimer (1062.4 Å^2^), and the PMM2 homodimer (998.9 Å^2^). As in the case of crystal structures, the interface region was rich in hydrophobic and polar contacts: the PISA analysis identified 14 hydrogen bonds and 10 salt bridges for the PMM2 homodimer, 18 hydrogen bonds and 12 salt bridges for the PMM1 homodimer, and 16 hydrogen bonds and 11 salt bridges for the PMM2/PMM1 heterodimer. The PISA analysis reported $\Delta^{i}G$ values ranging from –5.1 kcal/mol for the PMM2 homodimer to –5.8 kcal/mol for the PMM1 homodimer, with the PMM2/PMM1 heterodimer showing an intermediate $\Delta^{i}G$ of –5.2 kcal/mol.

PRODIGY, used as an independent estimate of binding free energy, returned ΔG values of –11.6 kcal/mol for the PMM2 homodimer, –12.5 kcal/mol for the PMM1 homodimer, and –12.1 kcal/mol for the PMM2/PMM1 heterodimer. The predicted dissociation constants were in the low nanomolar range (Kd = $3.1\cdot 10^{-9}$ M, $7.3\cdot 10^{-10}$ M, and $1.4\cdot 10^{-9}$ M, respectively), suggesting tight binding. Both tools consistently ranked the PMM1 homodimer as slightly more stable, but the differences across the three configurations were modest. Taken altogether, these results support the structural viability of PMM2/PMM1 heterodimers and suggest that they may form under physiological conditions. On the one hand, the consensus between tools that use different paradigms—PISA, which is physics-based and models thermodynamic stability, and PRODIGY, which is statistical and contact-based—supports the plausibility of the heterodimer. On the other hand, given the fact that the results are so similar, and considering the known variability of these tools, the results can only be interpreted in a comparative sense and are subject to structural sensitivity and approximation errors. Our ΔG values should therefore be viewed as qualitative indicators of relative interface stability rather than precise thermodynamic measurements.

**Figure 5:**
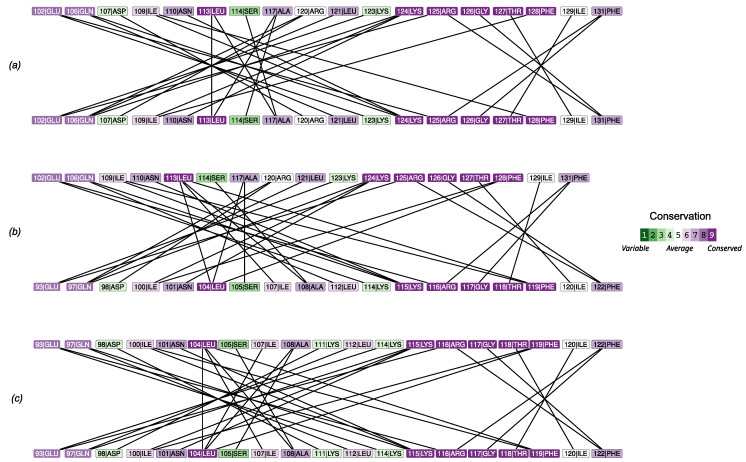
Analysis of contacts at the interface for the PMM1 homodimer (**a**), the PMM1/PMM2 heterodimer (**b**), and the PMM2 homodimer (**c**).

The analysis of amino acids’ interactions in homo- and heterodimers highlighted the quantitative strength of the interactions and the uniformity of bond distribution across the interface (Figure 5), in line with the results described above. In particular, two interaction clusters formed in all cases: a major (i.e., more extended) one involving an α-helix (102–128 in PMM1 and 93–109 in PMM2) and a minor (i.e., less extended) one involving the interaction of a more conserved stretch (five of six residues with conservation higher than seven) of β-strand from a subunit and a coil from the other (125–131 in PMM1 and 116–122 in PMM2).

### 2.4. Impact of Interface Mutations on PMM2 Dimer Stability

Missense variants of PMM2 mapping to the dimer interface were retrieved from ClinVar and gnomAD and mapped onto the structural model. Notably, several well-characterized pathogenic variants (e.g., p.Phe119Leu) fell within or near the interface region.

**Figure 6:**
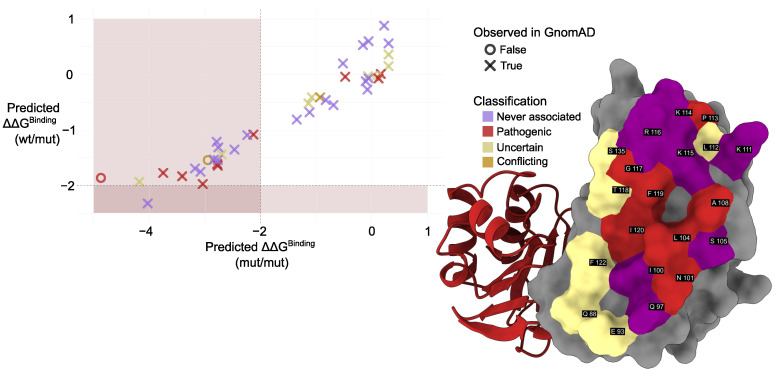
**Left**: Comparison of predicted $\Delta \Delta G^{\mathrm{binding}}$ values calculated using mmCSM-PPI. Colors indicate mutation classification while marker shape denotes presence (×) or absence (∘) of each variant in population datasets (gnomAD). Muted red shading marks the region of significant destabilization ($|\Delta \Delta G^{\mathrm{binding}}|$ > 2 kcal/mol, [18]). **Right**: Structural representation of the PMM2 dimer interface. Interface residues are shown as surfaces, colored according to their clinical annotation status in ClinVar, labeled based on UniProt residue numbering. Residues are classified as follows: never associated (not reported in ClinVar), pathogenic (at least one variant at that position is labeled pathogenic), and uncertain (only variants of uncertain significance reported). Coloring reflects these categories as described in the legend.

We used mmCSM-PPI to evaluate the impact of interface mutations found in gnomAD (Figure 6; Table A1 and Table A2). The predicted change in binding free energy (ΔΔG) upon mutation was calculated for the PMM2 homodimer with both subunits mutated (mut/mut) and the PMM2 homodimer with one mutated subunit and one wild-type subunit (mut/wt). p.Gly117Arg showed the most destabilizing effect (predicted $\Delta \Delta G^{\mathrm{binding}}$ = –4.87 kcal/mol), followed by p.Ile120Asn (−4.18) and p.Lys115Thr (−4.03). The destabilizing trend was conserved in both (mut/mut) and (mut/wt) configurations, although $|\Delta \Delta G^{\mathrm{binding}}|$ never exceeded 2 kcal/mol. Predictive modeling also identified potentially pathogenic variants not yet annotated in clinical databases. Notably, these variants are extremely rare in the general population ($\mathrm{MAF}<1\times 10^{-7})$. For example, p.Lys115Thr—reported in gnomAD but absent from ClinVar—exhibited a ΔΔG comparable to known disease-associated mutations, suggesting a destabilizing impact on the dimer interface. Interestingly, p.Lys115Thr was the only mutation exhibiting a significant destabilization in the wt/mut dimer, a condition that does not usually result in a clinical phenotype, not even in the presence of active site mutations, as in the case of p.Arg141His, but slightly widespread in the common population in heterozygosis with the wt allele frequency of $4.9\times 10^{-3}$.

The comparison of predicted $\Delta \Delta G^{\mathrm{binding}}$ with the conservation in the amino acids involved in the contact at the interface (Figure 5) revealed a lower $\Delta \Delta G^{\mathrm{binding}}$ in the most conserved amino acids.

Next, we performed a prediction of changes in binding (ΔΔG) for the PMM1/PMM2 heterodimer with mutations in the PMM2-interface amino acids (Table A2). Surprisingly, mutations that resulted in the destabilization of PMM2 mutant homodimer did not destabilize the PMM1/PMM2 heterodimer, except for p.Lys115Thr.

Additionally, we predicted the change in binding free energy (ΔΔG) in PMM2 mixed dimers, i.e., both subunits mutated (mut/mut) with two different mutations, selecting the possible mixed dimers from a recently published comprehensive literature review [19] and selecting those carrying at least one mutation in the dimerization interface (Table A3). Interestingly, all of the mutations with a predicted ΔΔG binding < 2 kcal/mol, resulted in a ΔΔG binding increase (>2 kcal/mol) when tested as mixed dimers with different mutations. The only exception was the mixed dimer p.Ile120Thr/Val231Met, which showed a ΔΔG binding −2.02 kcal/mol, consistent with previously published experimental data highlighting p.Val231Met as a highly destabilizing missense mutation [20].

Finally, we leveraged the AlphaFold3 server to explore the effect that mutating interface residues had on the interface, by modeling all gnomAD mutations considered previously. We found that out of the 41 mutations considered, 14 (approximately one-third) had no confident interacting pairs, while 4 fell within the wild-type model range (Figure S1a). We then examined the model structures and observed that AlphaFold consistently predicted dimers, regardless of the confidence level (Figure S1b). The only noticeable macroscopic structural effect was that one of the two subunits tended to exhibit moderate fluctuation across the mutants (Figure S1c). These results highlight how the combined use of multiple computational prediction tools (e.g., PISA, PRODIGY, ConSurf) can uncover overlooked variants with possible clinical relevance. These findings support the hypothesis that interface integrity is critical for proper dimerization and that pathogenic mutations may disrupt complex stability as a disease mechanism.

## 3. Discussion

The aim of this study was to evaluate the structural plausibility and relative stability of PMM2/PMM1 heterodimers compared to PMM2 and PMM1 homodimers. The dimerization of PMMs is tightly linked to their catalytic function, stability, and regulation. The formation of the dimer is required for full enzymatic activity [6]. Since PMM2 functions as an obligate dimer and its deficiency is the primary cause of PMM2-CDG [1], understanding the stability and compatibility of different dimeric configurations is relevant to exploring disease mechanisms. In particular, the inability of PMM1 to compensate for PMM2 loss in patients with PMM2-CDG raises questions about the physiological role of PMM1 and the potential interactions between the two paralogs, especially in compound heterozygous contexts [21]. To better understand the regulatory and functional mechanisms of PMM1 and PMM2, we explored the hypothesis that these proteins could form heterodimers under physiological conditions. Dimerization specificity is likely influenced by structural differences at the dimer interface, where key residues may favor the recognition and assembly of identical subunits rather than heterologous ones. To investigate this, we analyzed the stability and binding affinity of different dimeric combinations (PMM1 homodimer, PMM2 homodimer, and PMM1/PMM2 heterodimer) using structural modeling and interface analysis.

The co-expression analysis of PMM2 and PMM1 in multiple human tissues, including brain tissues—the most impacted by PMM2-CDG—supported the hypothesis that heterodimers could form *in vivo*. According to the GTEx data, PMM1 was generally more expressed, but PMM2 levels were not negligible in relevant tissues. These observations are in contrast with the inability of PMM1 to compensate for PMM2 deficiency in PMM2-CDG patients and point at a PMM1 major role in different pathways. Recently, a *PMM1* knock-out performed in fibroblasts derived from PMM2-CDG patients led to phenotypical improvement, strengthening this hypothesis [22]. The rationale behind this evidence is not yet fully understood, and it involves the bisphosphatase activity of PMM1. An interesting additional interpretation would be that the relative abundance and stoichiometry of the two different monomers could govern the shift between homo- and heterodimers. Notably, the GTEx data showed that PMM1 expression in regions such as the cortex, frontal cortex, amygdala, substantia nigra, cerebellar hemisphere, and cerebellum did not significantly correlate with PMM2, suggesting a spatial or cell-type specificity in their potential co-regulation. We attempted to validate tissue-level co-expression of PMM1 and PMM2 using SpatialDB, the Human Reference Atlas Functional Tissue Unit Explorer, and the Allen Brain Atlas. However, none of these platforms included the specific tissues where we observed co-expression in bulk transcriptomic data, and in the available tissues, PMM1 and PMM2 did not appear to co-localize. Consequently, we retained GTEx and Human Protein Atlas as our primary sources for general co-expression patterns, while recognizing that future spatial or single-cell studies with deeper coverage will be necessary to resolve cellular co-localization.

Overall, AlphaFold3-predicted dimers displayed well-packed, symmetrical interfaces with extensive residue engagement and hydrogen bonding, supporting their use as structurally plausible models for homodimers and heterodimers’ analyses. It is worth noting that the weaker $\Delta^{i}G$ for the crystallographic model may reflect incomplete biological assembly deposition or crystal packing artifacts. In contrast, AlphaFold3 predicted idealized symmetric dimers optimized for interface packing. These results suggest the structural viability and energetic plausibility of the PMM2/PMM1 heterodimer, supporting the idea that it can exist under physiological conditions. All of the three complexes showed extensive interface areas and a comparable number of hydrogen bonds and salt bridges, suggesting that heterodimer formation is energetically and geometrically plausible. The structural alignment of the AlphaFold3-predicted models with experimentally resolved crystal structures yielded RMSD values below 1.5 Å, validating the reliability of the predicted complexes. This high degree of structural similarity underscores the accuracy of the AlphaFold3 multimer predictions, providing confidence in their use to infer interface properties for the PMM2/PMM1 heterodimer in the absence of experimental structural data.

The interface analysis using PISA and PRODIGY produced consistent and complementary results. The differences in predicted binding energies across the three dimers were modest, suggesting that the heterodimer is energetically comparable to both homodimers. While PISA estimated a slightly higher $\Delta^{i}G$ for the PMM1 homodimer, PRODIGY ranked it as the most stable configuration, followed closely by the PMM2/PMM1 heterodimer. Despite slight methodological differences, both approaches converged on the idea that PMM2/PMM1 complexes are structurally and energetically viable.

The analysis of missense variants provided further insight into the structural determinants of dimer stability. Pathogenic variants of PMM2 have been extensively studied for their impact on both natural and synthetic ligand binding and their effects on enzymatic activity and protein stability. Most research has focused on ${\text{Glc-1},6\text{-}P}_{2}$, the phosphate group donor for PMM2, which acts as a key activator of the enzyme. ${\text{Glc-1},6\text{-}P}_{2}$ has also been implicated in stabilizing the PMM2 dimer, particularly in the presence of destabilizing mutations [1,12,20,23]. One particularly noteworthy finding concerns the most common disease-associated missense mutation, p.Arg141His, which results in a catalytically inactive enzyme that retains the ability to bind ${\text{Glc-1},6\text{-}P}_{2}$. Given its critical role in activation and structural stabilization, ${\text{Glc-1},6\text{-}P}_{2}$ has emerged as a promising therapeutic target for PMM2-CDG. Current efforts are focused on increasing its intracellular levels or identifying functional analogs that could mimic its beneficial effects [22,24,25]. In this paper, we performed an *in silico* analysis of missense variants that provided further insight into the structural determinants of dimer stability. Several pathogenic variants associated with PMM2-CDG were located at or near the dimer interface and were predicted to destabilize dimer formation. Notably, the p.Phe119Leu variant—among the most frequent pathogenic mutations—exhibited a strongly destabilizing effect in the mut/mut context, consistent with previous findings linking this variant to defective dimerization and destabilization in homozygous or compound heterozygous patients [1]. Predictive modeling using mmCSM-PPI revealed additional variants from population datasets (e.g., gnomAD) with ΔΔG values comparable to or more negative than known PMM2-CDG mutations. Notably, these variants are extremely rare in the general population. Among these, p.Lys115Thr emerged as a potentially pathogenic variant not yet reported in clinical databases (e.g., ClinVar). Interestingly, variants at the PMM2 homodimer interface that were predicted to destabilize dimer formation did not exhibit the same destabilizing effect on the formation of the PMM1/PMM2 heterodimer, with the exception of p.Lys115Thr. This unexpected result suggests that the ability of PMM2 variants to maintain heterodimerization with PMM1 may complicate the pathogenic mechanisms underlying PMM2-CDG, potentially influencing disease expression or severity in ways that remain to be fully understood. These findings point to the possibility that some destabilizing variants may be underrepresented in clinical annotations and support the hypothesis that impaired dimer stability is a key molecular mechanism in PMM2-CDG.

To further investigate the impact of mutations on interface qualities, we used the AlphaFold3 server to model the 41 interface mutants found in the general population (GnomAD) and in clinical cases (ClinVar). Our goal was not to assess pathogenicity or function but to observe whether certain regions of the structure appeared affected in AF3-generated models. When examining the relevance of residues across models and mutations, it became clear that the region 110–120, which was part of the major interaction cluster, was significantly affected in terms of confidence (orange patch region in Figure S1d). While this might suggest that interaction dynamics in this region are structurally sensitive to mutation, we caution that AlphaFold3’s reliability in detecting such effects is still being actively evaluated. Recent studies suggest AF3 may capture global conformational shifts [26], but it remains uncertain whether these predictions can be used to draw conclusions about stability or function at atomic resolution. Therefore, we report these findings purely descriptively and without linking them to pathogenicity or phenotypic outcomes. This observation suggests that the interaction dynamics in this region may play a critical role in the structural integrity of the protein and may be influenced by the mutations in ways that warrant further investigation.

Taken together, the results support a model in which dimer interface integrity is crucial for maintaining PMM2 enzymatic function. The formation of heterodimers with PMM1 appears structurally feasible and may contribute to dimer stability, but it may not be sufficient to restore activity in a pathological context. Although this study is limited to computational analyses and does not provide functional validation, the convergence of structural modeling, energetic binding estimation, and mutational impact analysis highlights the potential biological relevance of these findings. Given the use of predicted structures, the ΔG values reported here should be interpreted as relative estimates of interaction strength. The agreement between PISA and PRODIGY rankings supports the robustness of the observed trend, although experimental validation would be needed to confirm the binding affinity and functional implications of these interactions *in vivo*. While the absolute differences in predicted binding energies between dimer configurations are modest, they are consistent across independent tools (PISA and PRODIGY), with PMM1 homodimer consistently ranked as the most stable. Given the method’s inherent variability (∼1 kcal/mol), these differences may not indicate drastic functional divergence, but they do support the energetic plausibility of heterodimer formation.

## 4. Materials and Methods

### 4.1. Tissue Expression Analysis

TPM values for *PMM1* (ENSG00000100417.12) and *PMM2* (ENSG00000140650.13) were extracted from the GTEx (https://gtexportal.org/home/, [27]) v10 dataset [28] and merged with the sample metadata [29] to generate a sample-by-gene expression matrix. For each tissue, median TPM values were computed for both genes. Tissues were considered to support co-expression when both genes had median TPM > 10. Additionally, Spearman correlation coefficients were calculated between PMM1 and PMM2 across individual samples within each tissue. *p*-Values were adjusted for multiple testing using the Benjamini–Hochberg method, and tissues with adjusted *p*-values $p_{\mathrm{adj}}\leq 0.05$ and correlation coefficients ρ>0.5 were considered to exhibit significant co-expression. To assess protein-level co-occurrence, immunohistochemistry-based protein data for PMM1 and PMM2 [30] were retrieved from the Human Protein Atlas (HPA v24, https://www.proteinatlas.org/, [31]). Data were filtered to include only samples with detection levels classified as “Medium” or “High”. Co-occurrence was defined as the presence of both proteins in the same tissue and cell type. Due to the prevalence of “Uncertain” reliability scores for PMM1, and the use of HPA primarily as a source of supporting evidence for GTEx, both “Uncertain” and “Approved” entries were retained. A manual mapping was performed to align HPA and GTEx tissue nomenclature, allowing GTEx tissues to be flagged for confirmed protein-level co-detection based on this mapping. To visualize the results, we produced a scatter plot of median TPM values for PMM1 and PMM2 per tissue and annotated it with the HPA co-detection status. All analyses were conducted in R (v4.4.3) using packages from the tidyverse [32], along with data.table [33], ggrepel [34], and ggforce [35] for visualization and annotation. Scripts are available as part of Supplementary Materials.

### 4.2. Structural Modeling of Dimeric Complexes

Three dimeric configurations were modeled: PMM2 homodimer, PMM1 homodimer, and PMM2/PMM1 heterodimer. Full-length amino acid sequences of human PMM1 (UniProt ID: Q92871) and PMM2 (UniProt ID: O15305) were retrieved from UniProt (https://www.uniprot.org/ [36]). Structural models were generated using the AlphaFold3 (AF3) server (https://alphafoldserver.com, [37,38]) using the aforementioned sequences, leaving defaults without any ion or cofactor. Model confidence was evaluated using inter-chain predicted TM-scores (iPTM), per-chain predicted TM-scores (pTM), and the minimum predicted aligned error (PAE) between chains, extracted from the model summary output. Interface quality was primarily assessed through chain-pair iPTM values and inter-chain PAE, with iPTM > 0.8 and PAE < 1 Å considered indicative of high-confidence interfaces. All models were inspected for stereochemical quality and interface plausibility using ChimeraX V 1.10 [39] for visualization and comparison with available crystal structures (PDB: 7O4G for PMM2, 2FUC for PMM1).

### 4.3. Dimer Interface Analysis

The energetic and structural properties of the dimer interfaces were assessed using the Protein Interfaces, Surfaces, and Assemblies (PISA) tool from the European Bioinformatics Institute (https://www.ebi.ac.uk/pdbe/pisa/, [40,41]) leaving defaults. We used the AF3 outputs as coordinate files after mmcif -> pdb conversion through the pdbj conversion service [42]. For each model, PISA calculated the interface area, number of interacting residues, solvation free energy gain ($\Delta^{i}G$), hydrogen bonds, and salt bridges. These parameters were used to compare the relative stability of the homodimers and the heterodimer. Complementary to PISA, the PRODIGY web server (https://rascar.science.uu.nl/prodigy/, (accessed on 6 November 2024) [43,44]) was employed to estimate binding free energies (ΔG) and dissociation constants (Kd) for each complex, based on empirical inter-residue contact models. Calculations were performed at 25 °C using default settings, and results were used to benchmark relative interface stability across the homodimers and the heterodimers. To assess the conservation of the dimer interface, we generated residue–residue interaction networks for PMM1, PMM2, and PMM2/PMM1 heterodimers using ChimeraX. Intermolecular contacts were identified using the command contacts ifaces intramol false sel true reveal true, selecting only hydrogen bonds (O–N or N–O atom pairs within 3.5 Å), hydrophobic contacts (C–C atom pairs), and salt bridges (specific charged side-chain interactions). The resulting interaction networks were constructed in R using the tidygraph [45], ggraph [46], and tidyverse [32] packages, with residues as nodes and contacts as edges. Conservation scores were mapped onto the networks based on ConSurf (https://consurf.tau.ac.il/consurf_index.php [47,48]) analyses, using the 2FUC crystal structure for PMM1 and the 7O4G crystal structure for PMM2. Visualization emphasized chain identity and residue conservation using customized layouts.

### 4.4. Variant Curation and Annotation

The reference amino acid sequence for human PMM2 (UniProt ID: O15305) was retrieved and converted into a residue-by-residue table using R and the tidyverse package. Structural annotations were extracted from the UniProt GFF file, and only single-residue entries (where start and end positions are the same) were included. A list of interface residues was created using ChimeraX based on AlphaFold and PDB structural models (PDBs 2AMY, 7O0C, 7O1B, 7O4G) and integrated in the residue-by-residue table.

ClinVar data (https://www.ncbi.nlm.nih.gov/clinvar/ [49]) were used to identify missense variants associated with PMM2 [50] and integrated in the table by matching position and reference aa. Variants labeled as “uncertain significance” or with “conflicting interpretations” were excluded from pathogenicity-focused analyses, including those comparing interface residue effects and evaluating predictive model performance. Population variant data for PMM2 (ENSG00000140650) [51] were collected from gnomAD 4.1.0 (https://gnomad.broadinstitute.org/ [52]). The variant list was filtered to include only missense variants (based on the VEP_annotation column). ProtVar data (https://www.ebi.ac.uk/ProtVar/ [53]) associated with the PMM2 UniProt ID (O15305) were retrieved [54] and used to obtain population-level variant observations along with predictive scores from FoldX (ΔΔG) (https://foldxsuite.crg.eu/ (accessed on 6 November 2024)) and conservation. These scores were extracted and parsed from the structured ProtVar output files.

### 4.5. Effect of Interface Variants on Dimer Stability

Variants located at structurally mapped interface positions were extracted and reformatted into the required input syntax (e.g., A:p.Phe119Leu) using the results of the structural annotation pipeline. To assess the effect of interface variants on dimer stability, the mmCSM-PPI web server (https://biosig.lab.uq.edu.au/mmcsm_ppi/ [44,55]) was used. For each variant, the change in binding free energy (ΔG, kcal/mol) was computed for the PMM2 homodimer with both subunits mutated (mut/mut) and the PMM2 homodimer with one mutated subunit and one wild-type subunit (mut/wt). Negative ΔΔG values were interpreted as destabilizing, with values below −2.0 kcal/mol considered extremely destabilizing [18]. Mutations were then categorized based on their predicted impact and discussed in the context of known pathogenicity. Visualizations were created in R using ggplot2. Violin plots were used to compare the distribution of conservation and computational scores across unclassified variants. Scatter plots were generated to compare mmCSM-PPI predictions between homodimeric (mut/mut) and heterodimeric (mut/wt) contexts. Points were color-coded by clinical classification and shaped according to whether the variant was observed in the gnomAD dataset [52].

## 5. Conclusions

This study provided a computational assessment of the structural and energetic properties of PMM2 and PMM1 dimerization, with a focus on the plausibility and stability of PMM2/PMM1 heterodimers. Structural modeling with AlphaFold3, combined with an interface analysis using PISA and PRODIGY, suggests that heterodimers are energetically viable and display interface features comparable to those of the homodimers. The expression data further support the potential for heterodimer formation in physiologically relevant tissues, including fibroblasts. Moreover, the analysis of pathogenic variants at the PMM2 dimer interface highlights the role of interface integrity in modulating dimer stability and potentially contributing to disease severity.

While structural modeling supports the energetic viability of PMM2/PMM1 heterodimers, additional biochemical or cell-based studies are needed to confirm whether such complexes form or function under physiological conditions. While experimental validation remains necessary to confirm the functional outcomes of these findings, the consistency of results across structural, energetic, and mutational analyses strengthens the hypothesis that altered dimerization represents a key mechanism in the pathogenesis of PMM2-CDG. These findings contribute to a better understanding of PMM2 paralog interaction and may inform future studies on protein complex stability and targeted therapeutic strategies.

## Data Availability

The original contributions presented in this study are included in the article/Supplementary Material. Further inquiries can be directed to the corresponding author.

## Supplementary Materials

The following supporting information can be downloaded at https://www.mdpi.com/article/10.3390/molecules30122599/s1, Supplementary Figure S1: Modeling of PMM2 interface variants observed in ClinVar/gnomAD and related methods.

## Abbreviations

The following abbreviations are used in this manuscript:

CDGs	Congenital Disorders of Glycosylation
Glc-1,6-P2	Glucose-1,6-bisphosphate
GTEx	The Genotype-Tissue Expression (GTEx) Portal
HPA	Human Protein Atlas
iPTM	interface Predicted Template Modeling
Man-1-P	Mannose-1-phosphate
Man-6-P	Mannose-6-phosphate
PAE	Predicted Aligned Error
PISA	PDBePISA (Proteins, Interfaces, Structures and Assemblies)
PMM1	Phosphomannomutase-1
PMM2	Phosphomannnomutase-2
PRODIGY	PROtein binDIng enerGY
RMSD	Root-Mean-Square Deviation
TPM	Transcripts Per Million (gene and transcript expression GTEx
	Portal’s unit of measure)

## Appendix A

**Table A1:** Summary of PMM2 interface amino acid variants present in gnomAD and/or ClinVar, their classifications, allele frequencies, and homozygote counts from gnomAD (general population). N.A. = never associated.

wt	Position	mut	Classification	Allele Frequency	Homozygote Count
Q	88	H	Uncertain	3.75 × 10^−6^	0
E	93	D	Uncertain	3.11 × 10^−6^	0
N	101	K	Pathogenic	1.24 × 10^−6^	0
L	104	V	Pathogenic	1.86 × 10^−6^	0
A	108	V	Pathogenic	4.84 × 10^−5^	0
L	112	V	Uncertain	4.97 × 10^−6^	0
P	113	T	Pathogenic	6.21 × 10^−7^	0
P	113	S	Uncertain	0	0
P	113	L	Pathogenic	1.80 × 10^−5^	0
G	117	R	Pathogenic	0	0
G	117	C	Conflicting	0	0
T	118	A	Uncertain	1.29 × 10^−6^	0
T	118	S	Conflicting	1.93 × 10^−6^	0
F	119	L	Pathogenic	3.52 × 10^−5^	0
F	119	S	Pathogenic	6.40 × 10^−7^	0
I	120	N	Uncertain	6.39 × 10^−7^	0
I	120	T	Pathogenic	4.41 × 10^−5^	0
I	120	M	Pathogenic	5.11 × 10^−6^	0
F	122	L	Uncertain	4.46 × 10^−6^	0
S	135	R	Uncertain	8.87 × 10^−6^	0
A	108	E	N.A.	1.24 × 10^−6^	0
A	108	S	N.A.	6.20 × 10^−7^	0
E	93	K	N.A.	6.22 × 10^−7^	0
F	122	V	N.A.	1.28 × 10^−6^	0
G	117	S	N.A.	1.29 × 10^−6^	0
I	120	L	N.A.	6.40 × 10^−7^	0
K	111	N	N.A.	1.24 × 10^−6^	0
K	114	E	N.A.	1.86 × 10^−6^	0
K	114	R	N.A.	6.21 × 10^−7^	0
K	115	T	N.A.	6.22 × 10^−7^	0
N	101	D	N.A.	6.20 × 10^−7^	0
N	101	S	N.A.	6.20 × 10^−7^	0
P	113	R	N.A.	6.21 × 10^−7^	0
R	116	K	N.A.	1.24 × 10^−6^	0
R	116	M	N.A.	6.22 × 10^−7^	0
R	116	T	N.A.	6.22 × 10^−7^	0
S	105	G	N.A.	6.20 × 10^−7^	0
S	105	N	N.A.	3.10 × 10^−6^	0
S	105	R	N.A.	1.24 × 10^−6^	0
S	135	N	N.A.	1.90 × 10^−6^	0
S	135	T	N.A.	6.34 × 10^−7^	0

**Table A2:** Mutations in interface residues of the PMM2 homodimer identified in ClinVar and gnomAD and analyzed using mmCSM-PPI.

Mutation A	PMM2 Homodimer ΔΔG (kcal/mol)	PMM1/PMM2 Heterodimer ΔΔG (kcal/mol)
A Q88H	0.3	0.01
A E93D	−2.24	0.49
A E93K	0.3	−1.43
A N101D	−0.52	−0.09
A N101K	−2.13	−1.16
A N101S	−1.12	−0.87
A L104V	−2.78	−1.55
A S105G	−1.35	−0.74
A S105N	−0.69	−0.39
A S105R	0.22	0.82
A A108E	−0.16	0.47
A A108S	−0.06	0.61
A A108V	−0.48	−0.21
A K111N	−0.11	−0.19
A L112V	−1.08	−0.4
A P113L	0.12	0.04
A P113R	−0.08	−0.25
A P113S	0.1	−0.04
A P113T	0.16	−0.06
A K114E	−2.79	−1.17
A K114R	−0.83	−0.76
A K115T	−4.03	−2.29
A R116K	−2.84	−1.61
A R116M	−2.77	−1.65
A R116T	−3.18	−1.75
A G117C	−2.95	−1.46
A G117R	−4.87	−1.6
A G117S	−2.76	−1.12
A T118A	−1.14	−0.59
A T118S	−0.93	−0.6
A F119L	−3.04	−1.94
A F119S	−3.75	−1.9
A I120L	−2.47	−1.2
A I120M	−2.77	−1.56
A I120N	−4.18	−1.83
A I120T	−3.41	−1.75
A F122L	−2.7	−1.48
A F122V	−3.08	−1.57
A S135N	0.3	0.63
A S135R	−0.07	0.02
A S135T	−0.06	−0.05

**Table A3:** Mutations in interface residues of the PMM2 homodimer identified in ClinVar and gnomAD and analyzed in literature-described mixed dimers using mmCSM-PPI.

Mutation A	Mutation B	ΔΔG (kcal/mol)
A P113L	B F157S	−0.43
A R141H	B A108V	−0.11
A I120T	B G228C	−1.7
A F119L	B R141H	−1.81
A I120T	B V231M	−2.02
A P113L	B R141H	0.09
A A108V	B R123Q	−0.47
A P113L	B T237M	−0.55
A L104V	B D12H	−1.29
