# Supplementary material for: In Silico Analysis of Phosphomannomutase-2 Dimer Interface Stability and Heterodimerization with Phosphomannomutase-1
Source: Molecules. 2025 Jun 15;30(12):2599. doi: 10.3390/molecules30122599 (PMC12195792; doi:10.3390/molecules30122599)
Supplement: Supplementary file 1 [file molecules-30-02599-s001.zip › molecules-3647737-supplementary.pdf]

Additional methods

Structural and mutational data were compiled from two primary sources: 1. interface metrics were derived from AlphaFold-Multimer predicted structures (41 mutants and three dimers) and processed using ChimeraX interface analysis tools. These outputs were consolidated in `interfaces.csv`; 2. Mutational effect predictions were obtained by subsetting the main dataset, which includes homozygous mutation mmCSP-ppi pathogenicity scores (see main text).

Data were processed in R (v4.5) using the `tidyverse` package for data manipulation and visualization. Interface metrics were extracted from the interface CSV and af3 diagnostic JSON files for all (n=220) models. For each mutant, the number of confident interacting residue pairs and residue-specific interaction details were extracted and cleaned. JSON files containing interface predicted TM-scores (ipTM) were parsed using `grep` and regular expressions to isolate relevant data, which were then merged based on filenames.

Data were visualized using ggplot2 through a scatter plot showing the number of confident residue-residue interactions per mutant across models , and a heatmap representing the distribution of confidently interacting residues across mutants.

Spearman correlation analysis was performed to assess the relationships between interface interaction metrics (number of confident pairs and ipTM scores) and the mmCSM-ppi scores. The correlation matrix and adjusted confidence intervals were calculated using the `psych::corr.test` function with multiple testing correction.

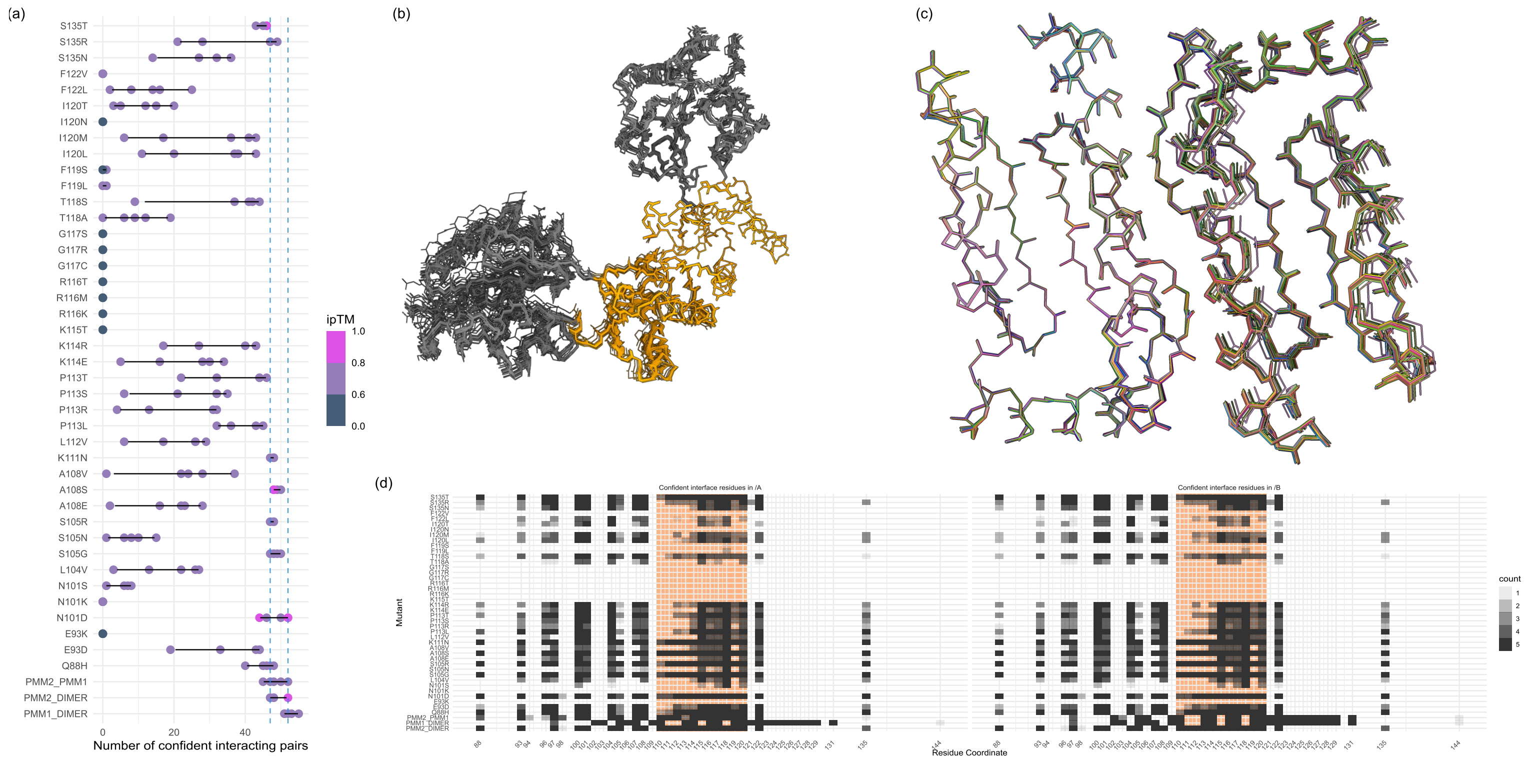

Figure S1: Modelling of PMM2 interface variants observed in ClinVar/Gnomad. a) Distribution of the number of confident interacting pairs across models. The three colros match ipTM diagnostic ranges ([0–0.6]: not reliable; [0.6–0.8]: uncertain;[0.8–1]: confident;) thin lines mark interquartile range of data; dashed blue lines mark ranges of wt PMM2 model; (b) structural superposition of all models to highest ranked PMM2 wt model (n=220, 5 replicates for 44 sequence pairs) based on the cap domain (highlighted in yellow); (c) zoom on the interface, coloured by model; (d) heatmap (faceted by chain) representing the distribution of confidently interacting residues across mutants. Count of interaction per residue is represented by tile opacity. The orange highlight mark residues falling in the interaction cluter 1 (see Main text and Fig. 6).
